# Effects of reduction technique for acute anterior shoulder dislocation without sedation or intra-articular pain management: a systematic review and meta-analysis

**DOI:** 10.1007/s00068-023-02242-8

**Published:** 2023-03-01

**Authors:** D. N. Baden, M. F. L. Visser, M. H. Roetman, D. P. J. Smeeing, R. M. Houwert, R. H. H. Groenwold, O. A. J. van der Meijden

**Affiliations:** 1 Emergency Physician, Diakonessenhuis, Utrecht, The Netherlands; 2 Medical Student, Amsterdam UMC, Amsterdam, The Netherlands; 3 Nurse Practitioner, Flevoziekenhuis, Almere, The Netherlands; 4 Trauma Surgeon in Training, UMC Utrecht, Utrecht, The Netherlands; 5 Trauma Surgeon, UMC Utrecht, Utrecht, The Netherlands; 6 Department of Clinical Epidemiology, Leiden University Medical Center, Leiden, The Netherlands; 7 Department of Biomedical Data Sciences, Leiden University Medical Center, Leiden, The Netherlands; 8 Orthopedic Surgeon, Albert Schweitzer Ziekenhuis, Dordrecht, The Netherlands

**Keywords:** Systematic review, Shoulder dislocation, Reduction success, Biomechanical reduction techniques, Leverage technique, Traction–countertraction technique

## Abstract

**Introduction:**

Anterior shoulder dislocations are commonly seen in the emergency department for which several closed reduction techniques exist. The aim of this systematic review is to identify the most successful principle of closed reduction techniques for an acute anterior shoulder dislocation in the emergency department without the use of sedation or intra-articular lidocaine injection.

**Methods:**

A literature search was conducted up to 15-08-2022 in the electronic databases of PubMed, Embase and CENTRAL for randomized and observational studies comparing two or more closed reduction techniques for anterior shoulder dislocations. Included techniques were grouped based on their main operating mechanism resulting in a traction–countertraction (TCT), leverage and biomechanical reduction technique (BRT) group. The primary outcome was success rate and secondary outcomes were reduction time and endured pain scores. Meta-analyses were conducted between reduction groups and for the primary outcome a network meta-analysis was performed.

**Results:**

A total of 3118 articles were screened on title and abstract, of which 9 were included, with a total of 987 patients. Success rates were 0.80 (95% CI 0.74; 0.85), 0.81 (95% CI 0.63; 0.92) and 0.80 (95% CI 0.56; 0.93) for BRT, leverage and TCT, respectively. No differences in success rates were observed between the three separate reduction groups. In the network meta-analysis, similar yet more precise effect estimates were found. However, in a post hoc analysis the BRT group was more successful than the combined leverage and TCT group with a relative risk of 1.33 (95% CI 1.19, 1.48).

**Conclusion:**

All included techniques showed good results with regard to success of reduction. The BRT might be the preferred technique for the reduction of an anterior shoulder dislocation, as patients experience the least pain and it results in the fastest reduction.

**Supplementary Information:**

The online version contains supplementary material available at 10.1007/s00068-023-02242-8.

## Introduction

Anterior shoulder dislocations are the most frequently seen large joint dislocations in the emergency department (ED) with an incidence close to 23 in 100.000 person-years [1, 2]. The dislocation is often the result of a sports injury or domestic falls [1, 3]. The age distribution has two peaks: one for men around 30 years and one for women around 50 years of age [2, 4]. Recurrence within 5 years of a shoulder dislocation occurs in 19–26% of the patients, most commonly in patients younger than 25 years old [2, 3].

In daily clinical practice, a wide variety of closed shoulder reduction techniques is being used, the choice of which seems to be determined by physician’s preference [5, 6]. In general, reduction techniques can be categorized based on their main principle being (1) traction, (2) leverage and (3) techniques based on biomechanical principles [7]. In a survey in 2003 among surgeons working in Dutch EDs, the Hippocratic (traction–countertraction), Kocher (leverage) and Stimson (traction–countertraction) techniques were the most frequently used [8]. In a repeat survey in 2016 among Dutch emergency physicians, the Hippocratic and Kocher technique were still frequently used [9]. However, also biomechanical techniques such as Milch [10] and Cunningham [11] were increasingly reported.

Most present studies did not directly compare multiple techniques, but instead describe the success rate of a single technique, which makes comparisons between techniques difficult [11–15]. Furthermore, in studies that compare reduction techniques, often different forms of sedation (mostly benzodiazepines) or intra-articular lidocaine injection (IAL) are applied, limiting the direct comparability between studies [16–19].

So far, no systematic review or meta-analysis has been conducted that compares the three groups of reduction techniques (traction–countertraction, leverage or biomechanical) comparing only the technique with exclusion of the use of sedation or IAL. Therefore, the aim of this study was to identify the most effective group of closed reduction techniques for an acute anterior shoulder dislocation without the use of sedation nor IAL in the emergency department.

## Materials and methods

### Eligibility criteria

Randomized and observational studies of patients 16 years and older with an acute anterior shoulder dislocation that compared two or more closed reduction techniques from a different principle of action were included. The reduction techniques had to be well defined, performed without the use of sedation (benzodiazepine, ketamine, propofol or etomidate), opiates in a more than normal analgesic dose or intra-articular pain management in the emergency department, and studies should compare the reduction success rates. Articles were included if written in English or Dutch. Excluded were letters, comments, abstracts for conferences, case reports, study protocols, reviews, biomechanical studies, animal studies or are non-hospital based (wilderness medicine, ski resorts) and non-comparative studies. The study protocol was not registered. This study was reported according to PRISMA guideline for systematic review 2020 (see appendix 4).

### Search strategy

The search query that was used is provided in Appendix 1. Three reviewers (DB, MV and MR) independently searched the PubMed (including MEDLINE), Embase and CENTRAL (Cochrane Central Register of Controlled Trials) electronic databases up to 15-08-2022**.** Disagreement regarding eligibility was resolved by discussion between the reviewers (DB, MV and MR).The identified records were first screened based on title and abstract and potentially suitable articles were read full text. The references of the included studies were screened for eligibility, and citation tracking was performed by using Web of Science to identify articles not found in the original search. In case no full-text version of the article was available, the corresponding authors were contacted by e-mail, and in case of no response, one reminder e-mail was sent.

### Data extraction

For each study, data extraction was performed independently by three reviewers (DB, MV and MR), after which the results were compared and discussed. There was no disagreement between reviewers. The following items were extracted: first author, year of publication, study design, country or countries in which the study was performed. In addition, the following information was extracted stratified by reduction technique: number of included patients, number of dislocations, proportion of female patients, mean age of included patients, dominant arm, pre-reduction fractures, primary dislocations, reduction success first reduction, reduction time, length of stay in the ED, Visual Analogue Scale (VAS) or Numeric (pain) Rating Scale (NRS), before, during and after reduction, and complications of the reduction.

### Classification of reduction techniques

A wide range of reduction techniques is described in the literature. Techniques can be classified based on their main principle of action: traction–countertraction (TCT—e.g., Hippocratic, Chair, Spaso, Matsen, Stimson, Davos, Traction—countertraction), leverage (e.g., Kocher, External rotation) or biomechanical reduction technique (BRT—e.g., Scapular manipulation technique, (modified) Milch, FARES, Cunningham) [5, 7].

### Outcome measures

The primary outcome was defined as the percentage of successful reductions in each of the three groups (TCT, leverage and BRT). The secondary outcome measures were time to reduction, ED length of stay, patient-reported pain score before, during and after reduction, and the number and type of complications.

### Quality assessment

Three reviewers (DB, MV and MR) assessed every article independently regarding the methodologic quality using the Methodological Index for Non-Randomized Studies (MINORS) [20]. The MINORS is a validated instrument for assessment of methodologic quality and reporting of observational studies of surgical interventions [20]. Further details on the MINORS criteria and scoring system are provided in Appendix 2. Disagreements were resolved by discussion between the reviewers (DB, MV and MR).

### Statistical analyses

Information about continuous outcome measures was converted to means and standard deviations when sufficient information was available using methods described in the Cochrane Handbook for Systematic Reviews of Interventions [21]. For each technique, the probability of treatment success was estimated using a random effects model that pooled information across different studies. For each technique, information was included directly from studies that reported on that technique. The function metaprop of the R package meta was used. For each pairwise comparison between reduction techniques, a meta-analysis was performed, which included only studies in which the relevant comparison was made. The computer program Review Manager (RevMan), version 5.4.1, was used [22]. All analyses were performed stratified by study design (i.e., RCTs and observational studies separately) as well as all study designs combined. Results of different studies were pooled by means of a random-effects model, using inverse variance weighting methods. In addition, for the primary outcome a network meta-analysis was performed in which the pairwise information is combined in a network that allows for simultaneous estimation of the effects of the three pairwise comparisons. The network meta-analysis was performed using the netmetabin function from the netmeta package in the statistical software package R.

For binary outcome measures, results are presented as risk ratios (RRs) with 95% confidence intervals (CIs). For continuous outcome measures, results are presented as differences in means with corresponding 95% CIs. Heterogeneity between studies was assessed by visual inspection of the forest plots and by estimating statistical measures for heterogeneity, that is, the Tau^2^ statistic and the Chi-square statistic. Inspection of a funnel plot of the primary outcome measure against its standard error was done to detect potential publication bias.

## Results

**Fig. 1:**
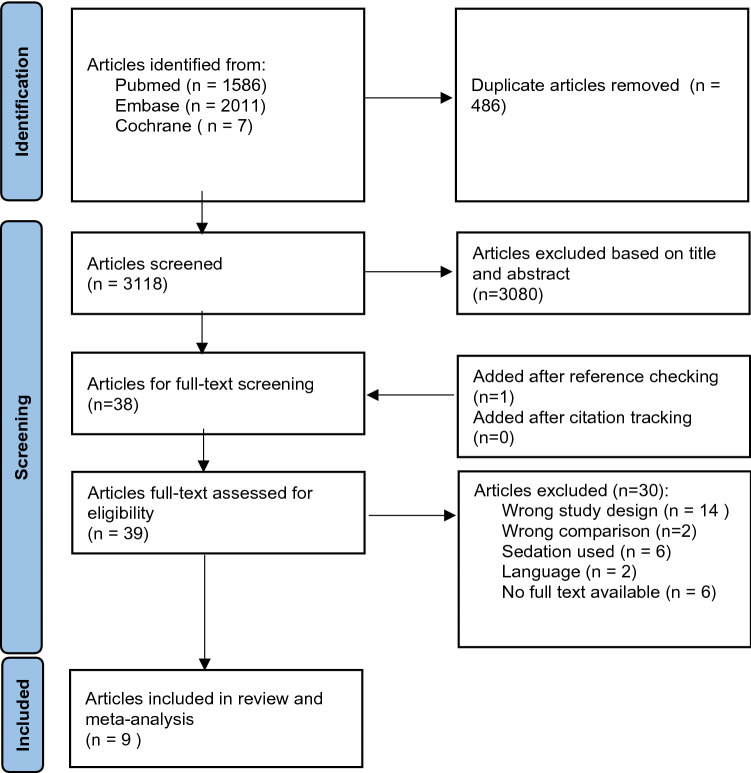
Flow diagram of selection of articles for meta-analysis

Figure 1 shows a flowchart of the literature search, which resulted in 9 studies [23–31] being included for review and meta-analysis. There were 5 RCTs, 3 prospective studies and 1 retrospective study. In the study of Guler et al. [26], four different techniques are compared, of which three were traction–countertraction. For the present study, the group treated with the Spaso technique was included in the meta-analysis, because this technique is also used in other studies. Inclusion of all three techniques would overrepresent the Guler study in the meta-analysis. For the secondary outcomes of ED length of stay and patient-reported pain score before and after reduction, there were too little data to perform a meta-analysis.

### Quality assessment

Regarding the different MINORS criteria, all studies had a maximum score for ‘clearly stated aim’ and ‘contemporary groups’, except for one. Regarding ‘unbiased assessment of the study endpoint’, only three studies scored one point and the other studies scored no points. Agreement with the MINORS criteria per study can be found in Appendix 3. The full MINORS criteria were not met by any of the included studies.

### Baseline characteristics of study participants

Information about the studies and characteristics of patients included in the meta-analysis is given in Table 1. The nine studies included in the meta-analysis comprised a total of 987 patients. There were 273 patients in the biomechanical group, 336 in the traction–countertraction group and 378 in the leverage group. The mean age was 38,6  years, and 315 of the 987 (31.9%) patients were female. The number of dislocations was the same as the number of patients included. Arm dominance was reported in two studies, pre-reduction fractures were reported in four studies and previous dislocations status was reported in three studies, so no comparison could be made for these outcomes [23, 25–28, 31].

**Table 1:** Characteristics of studies included in systematic review and meta-analysis of reduction techniques for shoulder dislocation

Study	Study design	Country	Treatment groups	Technique	No. of patients	Mean age yr	Female/male patients
Adhikari, 2018 [23]	PC	Nepal	Biomechanical	Scapular manipulation	23	36	4/19
			Leverage	External rotation	23	36	6/17
Amar, 2021 [27]	RCT	Israel	Biomechanical	Milch	35	44	9/26
			Traction–countertraction	Stimson	25	43	4/21
Beattie, 1986 [24]	PC	Scotland (UK)	Biomechanical	Milch	56	53	NR
			Leverage	Kocher	55	53	NR
Guler, 2015 [26]	RC	Turkey	Leverage	Kocher	40	34	9/31
			Traction–countertraction	Spaso	39	39	7/32
Maity, 2012 [28]	RCT	India	Biomechanical	FARES	80	37	15/65
			Leverage	External rotation	80	36	17/63
Rezende, 2015 [29]	RCT	Brazil	Leverage	Kocher	43	31	7/36
			Traction–countertraction	Spaso	45	30	6/39
Sapkota, 2015 [30]	RCT	Nepal	Biomechanical	Milch	26	27	9/26
			Leverage	External rotation	26	28	11/15
Sayegh, 2009 [31]	RCT	Greece	Biomechanical	FARES	53	41	10/43
			Traction–countertraction	Traction–countertraction	51	46	10/41
			Leverage	Kocher	50	44	13/37
Turturro, 2014 [25]	PC	Italy	Leverage	Kocher	61	40	15/46
			Traction–countertraction	Traction–countertraction	176	43	52/124

*RCT* randomized controlled trial, *PC* prospective cohort study, *RC* retrospective cohort study, *NR* not reported

### Reduction success

**Fig. 2:**
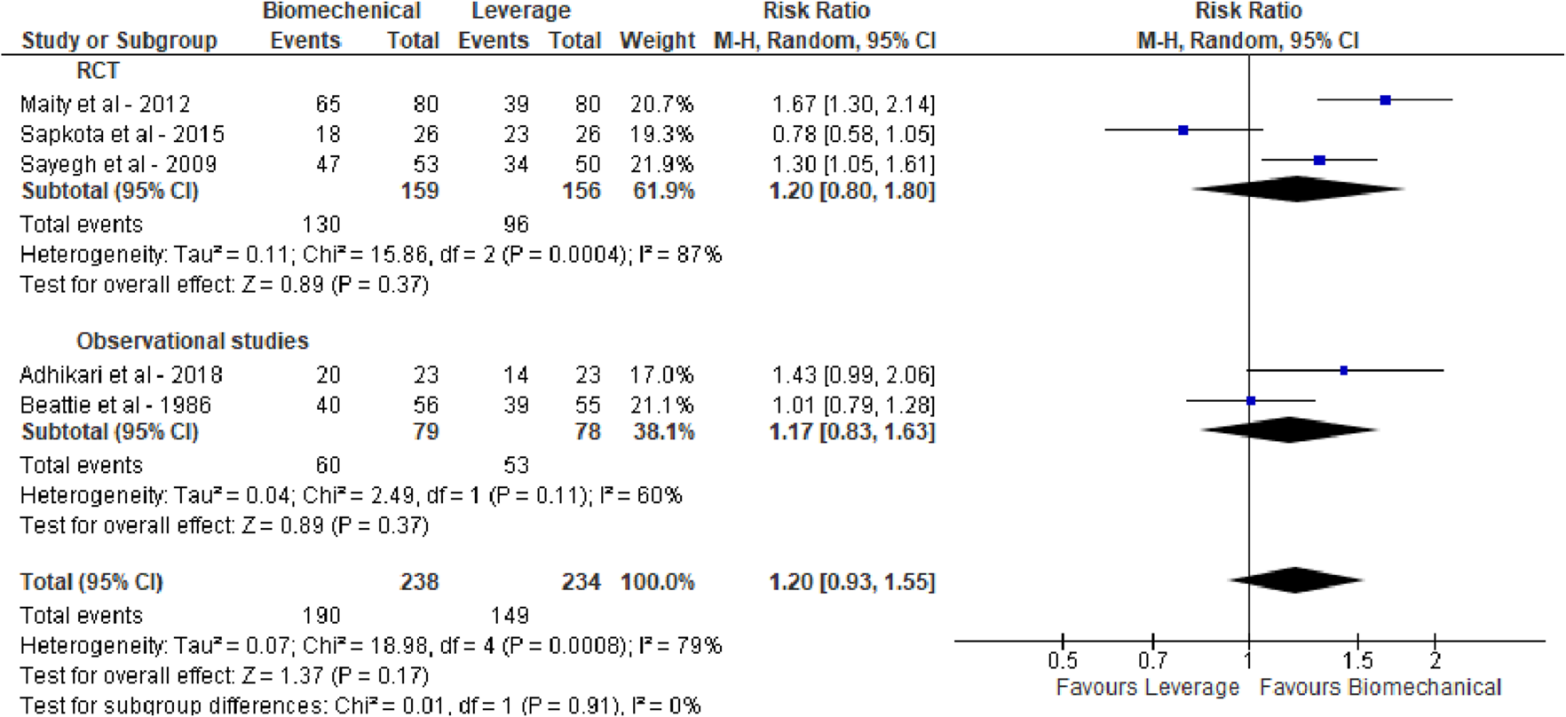
Reduction success of biomechanical versus leverage techniques for treatment of shoulder dislocation

**Fig. 3:**
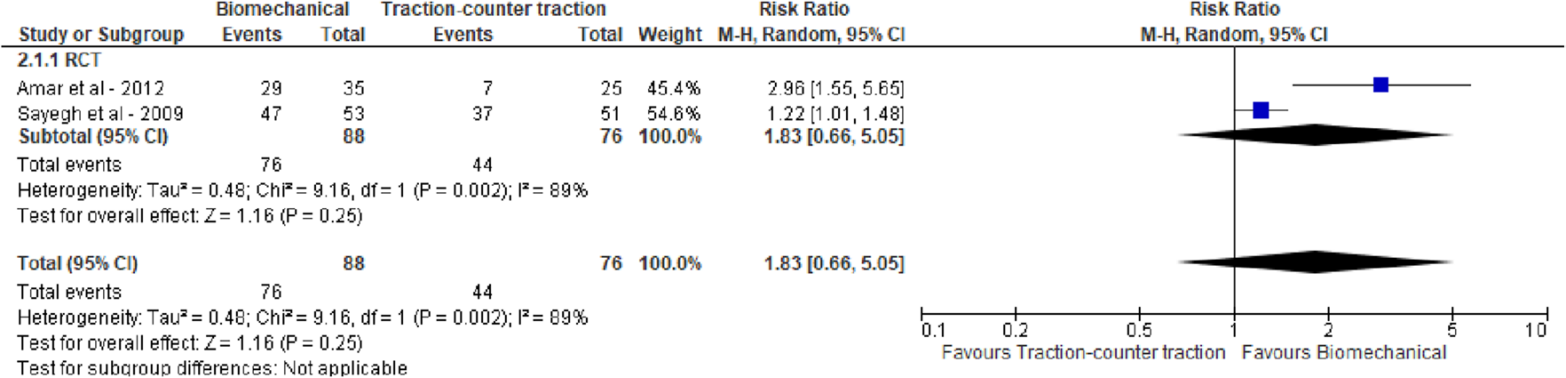
Reduction success of biomechanical versus traction–countertraction techniques for treatment of shoulder dislocation

**Fig. 4:**
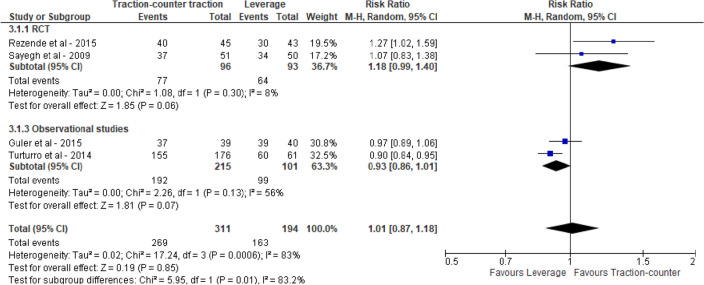
Reduction success of traction–countertraction versus leverage techniques for treatment of shoulder dislocation

For all three groups high success rates were reported, i.e., 0.80 (95% CI 0.74; 0.85), 0.81 (95% CI 0.63; 0.92) and 0.80 (95% CI 0.56; 0.93) for BRT, leverage and TCT, respectively. Pairwise meta-analysis of within-study comparisons between techniques did not reveal statistically significant differences in reduction success between the groups of reduction techniques: BRT vs leverage 1.20 (95% CI 0.93, 1.55), BRT vs TCT 1.83 (95% CI 0.66, 5.05) and TCT vs leverage 1.01 (95% CI 0.87, 1.18), see Figs. 2, 3 and 4. Meta-analysis stratified by study design did not lead to different conclusions.

In the network meta-analysis, similar yet more precise effect estimates were found: BRT vs leverage 1.25 (95% CI 1.05, 1.48), BRT vs TCT 1.28 (95% CI 1.04, 1.59) and TCT vs leverage 0.97 (95% CI 0.82, 1.15).

**Fig. 5:**
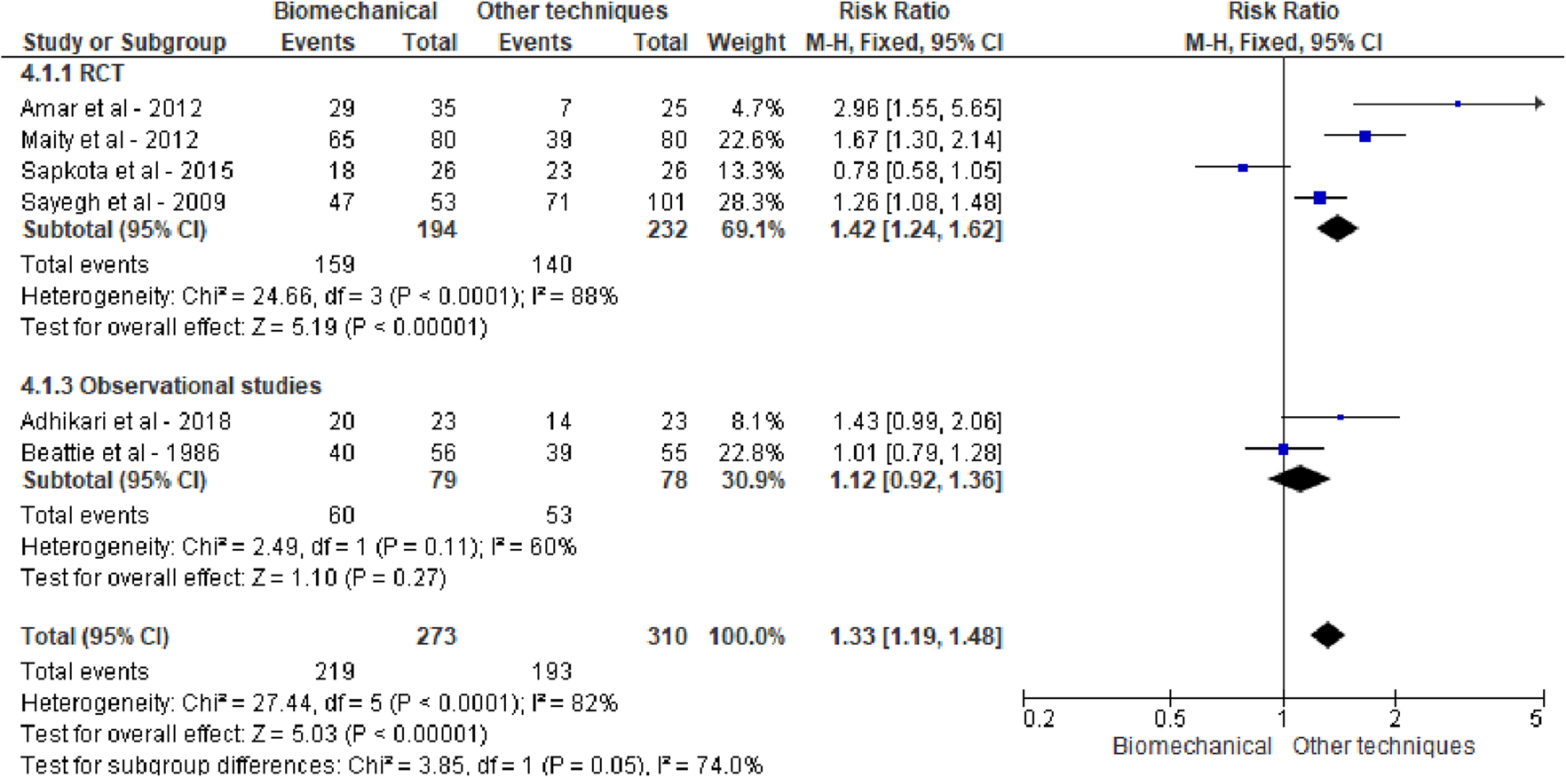
Reduction success of biomechanical versus either traction–countertraction or leverage techniques for treatment of shoulder dislocation

**Fig. 6:**
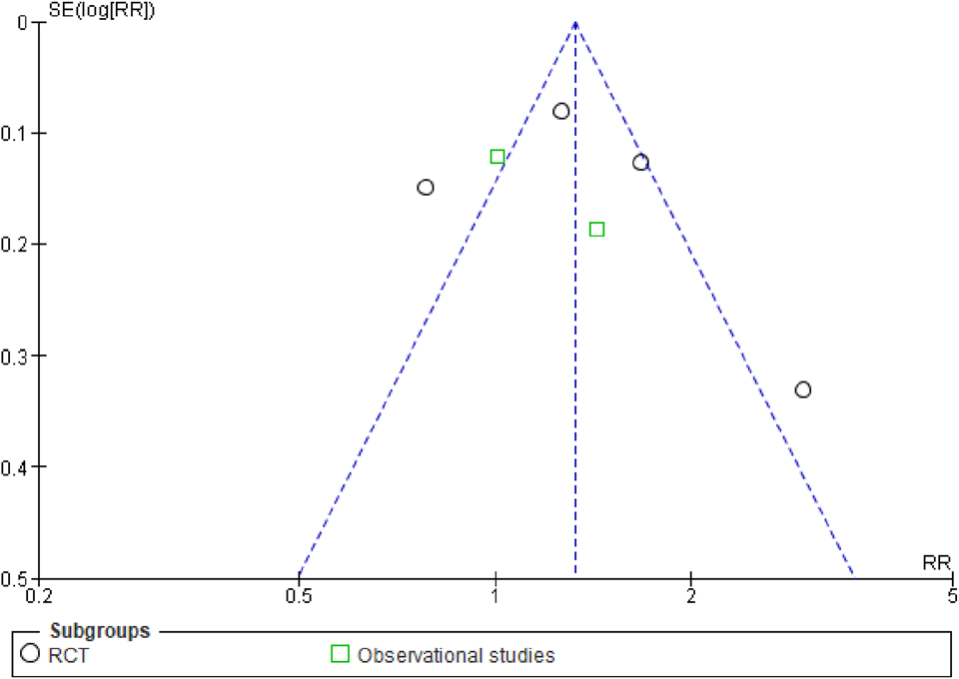
Funnel plot of reduction success biomechanical versus traction–countertraction and leverage

The relative risk of the comparison of leverage and TCT was only 1.01. Therefore, we did a post hoc analysis comparing the BRT group with the TCT and leverage groups combined, which showed a positive effect for BRT with a 33% increased probability of success, RR 1.33 (95% CI 1.19, 1.48), see Fig. 5. Again, stratification by study design did not change the results. The symmetry in the funnel plot in Fig. 6 did not reveal a possible publication bias.

### Patient-reported pain during reduction

All studies reported pain using a VAS score. Pooled results comparing reported pain were in favor of BRT versus leverage, with a difference in VAS of − 2.76 (95% CI − 4.16, − 1.36). In the BRT versus TCT, the difference in VAS was − 0.34 (95% CI − 0.61, − 0.08). Comparison between TCT versus leverage showed no difference in VAS 0.05 (95% CI − 0.25, 0.35), see Table 2.

**Table 2:** Pain experience by patients (VAS) during the treatment of a dislocated shoulder stratified by reduction technique

Comparison	Number of studies	Total number of participants	Mean difference in VAS (95% CI)
BRT vs leverage	3	309	− 2.76 (− 4.16, − 1.36)
BRT vs TCT	2	164	− 0.34 (− 0.61, − 0.08)
TCT vs leverage	3	268	0.05 (− 0.25, 0.35)

*BRT* biomechanical reduction technique, *TCT* traction–countertraction technique

### Reduction time

The time to reduction in the BRT group was 53 s faster compared to the leverage group (95% CI − 76, − 30). Between BRT versus TCT, this difference was 194 s (95% CI − 226, − 161). The time to reduction in the TCT group was 96 s faster compared to the leverage group (95% CI − 110, − 82), see Table 3.

**Table 3:** Difference in mean reduction time in the treatment of a dislocated shoulder stratified by reduction technique

Comparison	Number of studies	Total number of participants	Mean difference in seconds (95% CI)
BRT vs leverage	3	315	− 53 [− 76, − 30]
BRT vs TCT	2	164	− 194 [− 226, − 161]
TCT vs leverage	4	505	− 96 [− 110, − 82]

*BRT* biomechanical reduction technique, *TCT* traction–countertraction technique

### Complications

Just one complication was reported [24]. An 83-year-old lady suffered a spiral fracture of the humerus during reduction using the Kocher (leverage) technique.

## Discussion

The aim of this study was to compare reduction success of three groups of closed reduction techniques for acute anterior shoulder dislocation, applied at the emergency department and without the use of sedation or IAL. For all three groups, high success rates were reported; however, none of the individual groups of techniques was found to be superior, compared to the others.

In addition to success rate, several secondary outcomes were studied. Reduction was less painful in the BRT group when compared with both leverage (− 2.76, 95% CI − 4.16, − 1.36) and TCT (− 0.34, 95% CI − 0.61, − 0.08), where a difference in the MRS of 1.5 is considered clinically relevant [32, 33]. Furthermore, BRT was found to be a technique that required less time than both TCT and leverage. However, it is questionable whether a difference of 53 s is of clinical relevance; nonetheless, for patients experiencing enormous pain rapid relief could be valuable.

In the post hoc analysis, the BRT group was compared with a group in which the TCT and leverage groups combined. This analysis suggested that BRT is the best techniques for a successful and quick reduction of an anterior shoulder dislocation with the least reduction pain and with a low risk of complications.

The results of this meta-analysis are in agreement with the systematic review of Alkaduhimi et al., who also included studies with sedation and suggested that BRT (specifically SMT and FARES) are the best reduction techniques [6]. Also an earlier systematic review by Cunningham came to this conclusion [5]. In the meta-analysis by Dong et al. the conclusion was that almost all techniques seemed to have high success rates with low complication rates [34]. Dannenbaum et al. did a review where they concluded that there was no clear superior technique [35].

The difference between the current meta-analysis and the ones mentioned above is that we only included studies in which no advanced pain relief was used, such as sedation or IAL. Advanced pain relief techniques can ensure that the patient relaxes his musculature, and therefore, the technique used for the reposition is of minor importance. Nevertheless, advanced pain relief techniques themselves can pose a risk since there is less control during the reposition for consequences of stretching of vulnerable structures. Another difference between this meta-analysis and previous ones is that this study grouped the techniques by mode of action, making it possible to compare a larger number of studies.

A strength of this meta-analysis is that this study included similar studies, in which sedation or IAL was not used. Furthermore, all outcomes (e.g., success rate, pain and duration of techniques) that were considered are clinically relevant outcomes that are easy to interpret by treating physicians and by patients.

A limitation in this study is that the individual techniques could not be directly compared in separate meta-analyses, since for most separate techniques limited data were available. Therefore, this study does not provide evidence for the best individual technique; however, this study could give direction to the best group of techniques. A second possible limitation is that the MINORS criteria were used to assess the methodological quality of the included studies. To assess the methodological quality of RCTs, the Cochrane Risk of Bias tool is commonly used. However, since our study included both RCTs and observational studies, and we aimed to assess their methodological quality with the same tool, we opted for the MINORS tool. Slim et al. have externally validated the MINORS for RCTs and found it to differentiate well between different study designs, with randomized trials scoring higher than well-designed non-randomized trials. [20, 36, 37] Another limitation that the number of included studies was limited, thus also limiting the power to detect publication bias using the funnel plot depicted in Fig. 6. An additional limitation of this study is the limited number of studies included in each comparison and the heterogeneity in both the randomized and observational studies. Also, the wide range of years in which included studies were conducted may have influenced this study’s findings since emergency medicine has improved over the last years. However, again data were too limited to allow for separate analyses stratified by time period. Although this study’s outcome measures are of clinical importance, they may be of less relevance for individual patients. Length of stay (LOS) at the ED and total time to reduction possibly contribute more to a negative experience for patients, since quick reduction is suggested as the best way for quick pain relief [17, 27]. Additionally, prolongation of time to reduction could decrease success of used technique, possibly due to increase of muscle spasms [38]. LOS and total time to reduction were scarcely reported and were therefore not compared.

### Future research

Future research should focus on comparing individual BRT techniques in an RCT to discover the most effective and efficient closed reduction technique, preferably without the use of sedation and/or IAL. It could also be of interest to analyze a single technique and focus on the effectiveness and risk of a reduction both with and without the use of sedation, possibly providing clarity on the influence and the added value of sedation, given that sedation or IAL takes up time which increases length of stay in an increasing busy emergency department [39, 40]. Moreover, future research in individual techniques should include more outcomes that are directly of importance for patients such as LOS and time to reduction.

## Conclusion

In summary, almost all included techniques showed good results for reduction without sedation in the first attempt. This study might provide support that the BRT seems to be the preferred reduction technique in anterior shoulder dislocation, resulting in a rapid successful reduction with limited pain. Therefore, in daily practice and future research more focus should lie to the more patient-friendly and effective biomechanical reduction techniques.

## Supplementary Information

Below is the link to the electronic supplementary material.

Supplementary file1 (DOCX 14 KB)

Supplementary file2 (DOCX 15 KB)

Supplementary file3 (DOCX 16 KB)

Supplementary file4 (DOCX 32 KB)

## Data Availability

The dataset used and/or analyzed during the current study is available from the corresponding author on reasonable request.
