# Supplementary material for: Effects of reduction technique for acute anterior shoulder dislocation without sedation or intra-articular pain management: a systematic review and meta-analysis
Source: Eur J Trauma Emerg Surg. 2023 Mar 1;49(3):1383–92. doi: 10.1007/s00068-023-02242-8 (PMC10229450; doi:10.1007/s00068-023-02242-8)
Supplement: Supplementary file 1 — Supplementary file1 (DOCX 14 KB) [file 68_2023_2242_MOESM1_ESM.docx]

**Appendix 1:** Search query

*Pubmed: (n=1586*)

("anterior shoulder dislocation" OR "Shoulder Dislocation"[Mesh] OR forward shoulder luxation[Title/Abstract] OR forward glenohumeral dislocation[Title/Abstract] OR forward glenohumeral luxation[Title/Abstract] OR forward glenohumeral joint[Title/Abstract] OR forward shoulder dislocation[Title/Abstract] OR anterior shoulder dislocation[Title/Abstract] OR anterior shoulder luxation[Title/Abstract] OR anterior glenohumeral dislocation[Title/Abstract] OR anterior glenohumeral joint luxation[Title/Abstract] OR anterior glenohumeral luxation[Title/Abstract] OR anterior shoulder dislocation[Title/Abstract] OR anterior glenohumeral dislocation[Title/Abstract] OR ventral shoulder luxation[Title/Abstract] OR ventral glenohumeral dislocation[Title/Abstract] OR ventral glenohumeral joint luxation[Title/Abstract] OR ventral glenohumeral luxation[Title/Abstract] OR ventral shoulder dislocation[Title/Abstract] OR ventral glenohumeral dislocation[Title/Abstract])

**AND**

(treatment outcome[MeSH Terms] OR effectiveness, treatment[MeSH Terms] OR repositioning success rate[Title/Abstract] OR success rate[Title/Abstract] OR success[Title/Abstract] OR successful[Title/Abstract] OR successfulness[Title/Abstract] OR effectiveness[Title/Abstract] OR reduction technique[Title/Abstract] OR reduction method[Title/Abstract] OR reduce[Title/Abstract] OR relocat*[Title/Abstract] OR reposit*[Title/Abstract] OR repositioning techniques[Title/Abstract] OR biomechanical reposition techniques[Title/Abstract] OR leverage-based techniques[Title/Abstract] OR hippocratic method[Title/Abstract] OR kocher[Title/Abstract] OR acute shoulder reposition[Title/Abstract] OR scapular manipulation[Title/Abstract] OR closed reduction techniques[Title/Abstract] OR snowbird[Title/Abstract] OR cunningham[Title/Abstract] OR milch[Title/Abstract] OR eskimo technique[Title/Abstract] OR traction countertraction[Title/Abstract] OR Stimson[Title/Abstract] OR spaso[Title/Abstract] OR boss holzach matter[Title/Abstract])

-----------------------------

*Embase: (n=2011)*

1. shoulder AND dislocation
2. reduct*
3. reposit*
4. #2 OR #3
5. #1 AND #4
6. #5 AND 'human'/de AND 'article'/it

-----------------------------

*Cochrane: (n=7)*

Anterior shoulder dislocation
