# Supplementary material for: Effects of reduction technique for acute anterior shoulder dislocation without sedation or intra-articular pain management: a systematic review and meta-analysis
Source: Eur J Trauma Emerg Surg. 2023 Mar 1;49(3):1383–92. doi: 10.1007/s00068-023-02242-8 (PMC10229450; doi:10.1007/s00068-023-02242-8)
Supplement: Supplementary file 2 — Supplementary file2 (DOCX 15 KB) [file 68_2023_2242_MOESM2_ESM.docx]

**Appendix 2:** Adapted MINORS criteria

| **Methodological items** | **2** | **1** | **0** |
| --- | --- | --- | --- |
| **A clearly stated aim** | Aim or hypothesis including outcomes have been reported | Aim or hypothesis have been reported  without a clear outcome | Not reported |
| **Inclusion of consecutive patients** | Explicit inclusion and exclusion criteria have been reported | Unclear or poor description inclusion and exclusion criteria have been reported | Not reported |
| **Prospective collection of data Retrospective** | Prospective with a description of the protocol | Prospective without a description of the protocol | Retrospective |
| **Endpoints appropriated to the aim of the study** | Outcomes are appropriate to the aim of the study | Outcomes are not appropriate to the aim of | Not reported |
| **Unbiased assessment of the study endpoint** | Blind evaluation of objective outcomes | Reason not blinded stated | Not reported |
| **Follow-up period appropriate to the aim of the study** | Emergency department visit | - | Not reported |
| **Loss to follow up** | All inclusions are the results reported or the exclusions described | Not all inclusions are the results reported and without described | Not reported |
| **Prospective calculation of the study size** | Power analysis has been performed | Explanation for the number of included patients without a power analysis | Not reported or not performed |
| **An adequate control group** | Having a intervention recognized as the optimal  intervention according to the available published data | Not applicable | Not reported |
| **Contemporary groups** | Study group and controls have been managed during the same time period | Study group and controls have not been managed during the same time period | Not reported or unclear description |
| **Baseline equivalence of groups** | Adequate randomisation and description | Baseline characteristics have been described | Not reported |
| **Adequate statistical analyses** | Statistical analysis has been described including the type of test | Inadequate statistical analysis | Not reported |
