# Supplementary material for: Effects of reduction technique for acute anterior shoulder dislocation without sedation or intra-articular pain management: a systematic review and meta-analysis
Source: Eur J Trauma Emerg Surg. 2023 Mar 1;49(3):1383–92. doi: 10.1007/s00068-023-02242-8 (PMC10229450; doi:10.1007/s00068-023-02242-8)
Supplement: Supplementary file 3 — Supplementary file3 (DOCX 16 KB) [file 68_2023_2242_MOESM3_ESM.docx]

**Appendix 3:** MINORS score per included study

| **Studie** | Aim | Inclusion | Data | Endpoints | Unbiased | Follow-up | Loss to  follow-up | study  size | Adequate  control group | Contemporary  groups | Baseline  Equivalence  of groups | Adequate  statistical analyses | **Total** |
| --- | --- | --- | --- | --- | --- | --- | --- | --- | --- | --- | --- | --- | --- |
| Adhikari | 2 | 2 | 2 | 2 | 0 | 2 | 2 | 0 | 2 | 2 | 0 | 2 | **18** |
| Amar | 2 | 2 | 2 | 2 | 1 | 2 | 2 | 0 | 2 | 2 | 2 | 2 | **21** |
| Beattie | 1 | 1 | 1 | 2 | 0 | 2 | 2 | 0 | 2 | 2 | 0 | 0 | **13** |
| Guler | 2 | 1 | 0 | 1 | 0 | 2 | 1 | 2 | 1 | 1 | 1 | 2 | **14** |
| Maity | 2 | 2 | 1 | 2 | 0 | 2 | 1 | 2 | 2 | 2 | 2 | 2 | **20** |
| Rezende | 2 | 2 | 2 | 1 | 1 | 2 | 2 | 2 | 2 | 2 | 1 | 2 | **21** |
| Sapkota | 2 | 1 | 1 | 2 | 0 | 2 | 2 | 0 | 2 | 2 | 1 | 1 | **16** |
| Sayegh | 2 | 2 | 2 | 2 | 1 | 2 | 2 | 2 | 2 | 2 | 2 | 2 | **23** |
| Turturro | 2 | 1 | 2 | 2 | 0 | 2 | 2 | 0 | 1 | 2 | 1 | 2 | **17** |
